# Editorial on Psychological distress in the Greek general population during the first COVID-19 lockdown

**DOI:** 10.1192/bjo.2021.1014

**Published:** 2021-09-17

**Authors:** Samuel Tromans

**Affiliations:** Department of Psychiatry of Intellectual Disability, Leicestershire Partnership NHS Trust, UK;; Department of Health Sciences, University of Leicester, UK

**Keywords:** COVID-19, post-traumatic stress disorder, depressive disorders, anxiety disorders, epidemiology

## Abstract

This editorial discusses a study by Karaivazoglou and colleagues, where the authors investigated anxiety, depressive, post-traumatic stress-related symptoms and coronavirus disease 2019 (COVID-19)-related beliefs in the Greek population during the first COVID-19 lockdown, via an anonymous online survey.

Coronavirus disease 2019 (COVID-19), first recognised in December 2019, brought about a global pandemic of respiratory disease, presenting a huge international challenge for both healthcare services and the corresponding populations which they serve.^1^ As of 4 April 2021, there have been a total of 130 459 184 confirmed cases of COVID-19, with 2 842 325 deaths.^2^

In addition to having an impact on the physical health of the global population, pandemics can also have a substantial impact on mental health. This can be measured in a variety of ways, including numbers of new mental health service referrals,^3^ psychiatric hospital admissions^4^ and interview- and/or survey-based approaches.

Karaivazoglou and colleagues^5^ used an anonymous online survey-based approach to estimate the prevalence of symptoms relating to psychological distress in the Greek population during the first COVID-19 lockdown. This editorial discusses the study in further detail, and what can be learned from its findings.

## Methodology

The authors developed an online survey, promoted via both social media and press releases. This approach is likely to yield a sample not entirely representative of the overall Greek population, which the authors acknowledge, as social media promotion is likely to disproportionately reach younger, more highly educated adults.

The study time period was from 10 April to 4 May 2020, during which time the Greek population were under strict lockdown measures in order to minimise COVID-19 spread.

## Demographic details and medical history

The majority of survey respondents were female, representing 73% (*n* = 1052) of the total study population who completed the survey (*n* *=* 1443). The study population also appears skewed towards younger adults, with 78% (*n* *=* 1120) of respondents being aged between 18 and 50 years, as well as the more highly educated, as 59% (*n* = 855) reported having a PhD or other postgraduate degree.

With regards to regional representation among survey respondents, the Central Greece administrative region (population estimate^6^ 546 870) was overrepresented relative to its population size, comprising 64% (*n* = 930) of total respondents, with more populous regions such as Attica (population estimate^6^ 3 812 330) being comparatively underrepresented (18%; *n* *=* 258),

However, although the study population is unrepresentative of the overall Greek population, the authors collected relevant demographic data to provide a detailed description of the respondent group.

## COVID-19-related beliefs

The study assessed the participant's COVID-19-related beliefs and behaviours, using questions taken from the Standard Questionnaire on Risk Perception of an Infectious Disease Outbreak.^7^ The questions, originally in English, were translated into Greek by two independent translators. Accurate translation of questionnaires can prove challenging, with the translation process prone to error.

The majority of respondents (63.4%) considered COVID-19 to be a severe infection, and 57.6% were significantly worried about getting infected themselves. Furthermore, 89.3% of respondents considered the protective measures to be highly efficacious, with 98.3% reporting adherence with the protective measures. The views pertaining to the efficacy of protective measures are perhaps understandable, as the number of COVID-19 cases and deaths in Greece were low during the first few months of the pandemic. It would be interesting to observe how perceptions across the Greek population may have subsequently changed over the course of the pandemic, as COVID-19-related cases and deaths rose during the second wave in autumn 2020, and adherence with the protective measures may have proved more challenging with time, and in the case of some, associated economic hardship.^8^

## Post-traumatic stress

The authors measured post-traumatic stress relating to the pandemic using the Impact of Event Scale-Revised (IES-R).^9^ Based on IES-R scores, over a third of respondents (36.4%; *n* = 506) experienced COVID-19-related post-traumatic stress disorder (PTSD), with a further 8.7% (*n* *=* 121) having probable PTSD. However, such findings in isolation do not equate to a clinical diagnosis, and subsequent evaluation of participants by trained mental healthcare professionals would have been valuable in determining what proportion of participants met PTSD criteria based on clinical judgement and/or criteria derived from major diagnostic classification systems.

Following multivariate linear regression, independent predictors of increased post-traumatic stress symptoms included female gender (*P* < 0.001), having slight, enough, or great worry about COVID-19 infection and a perception that COVID-19 protective measures were probably not efficacious (*P* = 0.020). Protective factors included having a postgraduate degree (*P* *=* 0.003) or PhD (*P* = 0.003), having had no prior psychiatric treatment (*P* < 0.001) and not adhering to protective measures (*P* *=* 0.049).

The finding that non-adherence with protective measures appears to be associated with significantly reduced post-traumatic stress symptoms is consistent with those of another Greek study by Parlapani et al^10^ who reported that greater adherence with COVID-19 guidance was associated with greater levels of fear reported. The authors recommended that fear can be effective in bringing about adherence with public health measures in some individuals, but can bring about detrimental effects in others, such as denial or detrimental mental health consequences. This finding is also interrelated to the perceived efficacy of public health measures, as individuals viewing such measures as probably not efficacious experienced significantly greater levels of post-traumatic stress.

## Anxiety and depression

Clinical features relating to anxiety and depression were assessed using the Hospital Anxiety and Depression Scale (HADS).^11^ However, the HADS was originally intended to measure anxiety and depressive states within an out-patient medical clinic setting,^11^ and may behave differently when used to measure such states across the general population. Nevertheless, Michopoulos and colleagues^12^ previously found the Greek version of the HADS to be ‘acceptable, reliable and valid’ when comparing findings from hospital in-patients, out-patients and the general population.

Following multivariate linear regression analysis, factors independently predictive of anxiety scores included risk factors such as female gender (*P* <0.001), as well as increased perceived severity in case of COVID-19 infection (*P* *=* 0.015), and having slight (*P* *=* 0.003), enough (*P* <0.001) or great (*P* <0.001) worry about COVID-19. Protective factors included having a postgraduate degree (*P* = 0.002) or a PhD (*P* *=* 0.002) and having no prior history of psychiatric treatment (*P* *=* 0.005).

In comparison, risk factors for increased depressive scores included being married (*P* *=* 0.024), being divorced (*P* *=* 0.028), as well as increased perceived severity in the case of personal COVID-19 infection (level of severity 2 (*P* *=* 0.049); level of severity 3 (*P* *=* 0.018); level of severity 4 (*P* *=* 0.037); level of severity 5 (*P* *=* 0.072)). and enough (*P* *=* 0.019) or great (*P* < 0.001) worry about COVID-19. Protective factors included having a postgraduate degree (*P* = 0.001) or a PhD (*P* = 0.006).

Interestingly, having a postgraduate degree or PhD independently predicted reduced anxiety scores, depression scores and post-traumatic stress symptoms. The authors suggested that this may be because there may have been less of an impact on such individuals financially from lockdown measures. Indeed, there is evidence to suggest that jobs relating to knowledge-intensive services were less likely to be in sectors subjected to forced closure.^13^ Furthermore, a UK-based survey^14^ demonstrated that loss of income was associated with higher reported levels of anxiety, depression and trauma symptoms during the COVID-19 pandemic, albeit with data collection having taken place at a slightly earlier time period (23–28 March 2020) than for the present study.

## Conclusions

The reported findings of this study are suggestive of high rates of anxiety, depression and PTSD during the first COVID-19 lockdown in Greece. Such high rates of depression and anxiety are broadly consistent with findings from other studies across the world. This was demonstrated in a meta-analysis by Salari et al^15^ who reported a pooled prevalence estimates of 31.9% (95% CI 27.5–36.7) for anxiety (across 17 studies with a total sample size of 63 439), and 33.7% (95% CI 27.5–40.6) for depression (across 14 studies comprising a sample size of 44 531). However, one must interpret such findings with caution, as although the constituent measurements scales have value, they are not a substitute for clinical diagnosis. Furthermore, the study population is not representative of the overall Greek population, and, indeed, individuals with increased distress pertaining to the COVID-19 pandemic may be more likely to have engaged in such a study relative to their less distressed peers.

Nevertheless, the study provides a valuable insight into the self-reported mental state of a subgroup of the Greek population from April to May 2020, as well as risk factors associated with anxiety, depression and post-traumatic stress levels. Additionally, the demographic details of the population studied are well described.

Repeating such measures at different time points could help discern how the burden of anxiety, depression and PTSD evolve over the course of the pandemic. Furthermore, inviting a subgroup of the study population for clinical assessment by trained healthcare professionals could help establish how predictive measures such as the IES-R and HADS are in this particular context. If either or both measures are found to have good diagnostic accuracy as predictors of mental health diagnoses in this context, they could act as valuable tools to help identify some of the adults with greatest clinical need.
